# Open Access to Trials Register

**DOI:** 10.1371/journal.pmed.0020049

**Published:** 2005-02-22

**Authors:** Susanne McCabe

I find the arguments raised by the *PLoS Medicine* editors very useful [1] as I had not considered that a scientific community would tolerate barring access to registers of trials. It leaves huge gaps for exploitation by privileged groups.

It is not only colleagues in research and allied professions who need access but the global community, including members of the public wherever they live, those who participate in trials and those who will be on the receiving end of their outcomes.

The annual reports of research ethics committees (RECs) are supposedly in the public domain after approval by Strategic Health Authorities in the UK. But very few members of the public know of their existence or how to access them. Approaches to individual committees even now can meet with varied reactions, from suspicious, defensive, or hostile—reluctantly sending one report, quizzing as to which organisation the enquirer belongs to and why they should want one—to extremely welcoming of interest and discussion.

The annual reports should be easily accessible online by now, surely, but they are not. The activities of RECs and information on what research is being carried out in the name of society as a whole largely remain hidden from public view.

There is no information about public access on COREC (Central Office for Research Ethics Committees; www.corec.org.uk) or OREC (Office for Research Ethics Committees; www.orecni.org.uk). COREC has not been open about dealing with issues of concern raised with them in the past. They do state that public interest is welcome now, so it would show a real commitment to making research activity more open if they would show support for totally open access to a register and to promote that through their Web site.
